# Transcriptional response to West Nile virus infection in the zebra finch (*Taeniopygia guttata*)

**DOI:** 10.1098/rsos.170296

**Published:** 2017-06-28

**Authors:** Daniel J. Newhouse, Erik K. Hofmeister, Christopher N. Balakrishnan

**Affiliations:** 1 Department of Biology, East Carolina University, Greenville, NC 27858, USA; 2 US Geological Survey, National Wildlife Health Center, 6006 Schroeder Road, Madison, WI 53711, USA

**Keywords:** transcriptome, RNAseq, flavivirus, avian, immune response

## Abstract

West Nile virus (WNV) is a widespread arbovirus that imposes a significant cost to both human and wildlife health. WNV exists in a bird-mosquito transmission cycle in which passerine birds act as the primary reservoir host. As a public health concern, the mammalian immune response to WNV has been studied in detail. Little, however, is known about the avian immune response to WNV. Avian taxa show variable susceptibility to WNV and what drives this variation is unknown. Thus, to study the immune response to WNV in birds, we experimentally infected captive zebra finches (*Taeniopygia guttata*). Zebra finches provide a useful model, as like many natural avian hosts they are moderately susceptible to WNV and thus provide sufficient viremia to infect mosquitoes. We performed RNAseq in spleen tissue during peak viremia to provide an overview of the transcriptional response. In general, we find strong parallels with the mammalian immune response to WNV, including upregulation of five genes in the Rig-I-like receptor signalling pathway, and offer insights into avian-specific responses. Together with complementary immunological assays, we provide a model of the avian immune response to WNV and set the stage for future comparative studies among variably susceptible populations and species.

## 1. Introduction

West Nile virus (WNV) is a single-stranded RNA flavivirus that exists in an avian-mosquito transmission cycle, where birds (typically Passeriformes) act as the primary amplification hosts. In addition to birds, nearly 30 other non-avian vertebrate species have been documented as hosts [1]. Although many WNV-infected hosts are asymptomatic, WNV infection can cause severe meningitis or encephalitis in those that are highly susceptible. Avian species for the most part exhibit low to moderate susceptibility. That is, individuals become infected and develop sufficient viremia for transmission via mosquito blood meal, but the hosts recover and avoid significant mortality (reviewed in [2]). First described in 1937, WNV has not resulted in widespread avian decline throughout its historical range [3], perhaps due to host–parasite coevolution. However, the emergence of WNV in North America in 1999 has negatively impacted a wide range of populations [4,5]. Surveys of North American wild birds have shown a variety of competent WNV hosts, with varying degrees of susceptibility, morbidity and pathogenicity [2]. American robins (*Turdus migratorius*) appear to be the main host in spreading WNV infection in North America [6], but infection appears most detrimental to members of Family Corvidae [7]. Despite great variation in susceptibility, the mechanisms underlying this variation are primarily unknown [2].

Largely due to interest in human health implications, most work describing the host immune response to WNV infection has been performed in mammalian systems [8]. From these studies, we know that in mammals, both the innate and adaptive arms are critical for virus detection and clearance [9,10]. Within the innate immune response, the retinoic acid-inducible gene 1 (Rig-I)-like receptor (RLR) pathway appears to play a key role in viral clearance. This pathway recognizes viral products and initiates type I interferon expression [11]. Mice lacking the viral recognition RLR genes in this pathway, DDx58 (Rig-I) and IFIH1 (MDA5), become highly susceptible to WNV infection [12]. In the adaptive immune system, a broad range of components appear to play important roles in mounting a response, including antibody and CD4+ and CD8+ T cells [9,13,14]. Interestingly, major histocompatibility complex (MHC) class I genes are upregulated post-infection [15,16]. Viruses typically evade MHC class I detection [17,18], as MHC class I molecules bind and present viral peptides to CD8+ T cells. However, the purpose of WNV-induced MHC expression is unclear.

While the mammalian immune response to WNV infection has been extensively studied, the avian immune response remains mostly unknown. Of the studies in birds, many involve experimentally infecting wild caught birds (reviewed in [2]), or domestic chickens (*Gallus gallus*) [19]. These studies primarily focus on viral detection, tissue tropism, antibody production or lymphocyte counts [2,19,20]. Little is known about the molecular mechanisms driving the immune response to WNV infection (but see [21]). Furthermore, current avian WNV studies suffer many challenges. Wild caught birds may be co-infected with other parasites (e.g. avian malaria) and are difficult to maintain in captivity for experimental infection studies. Chickens, although an avian model species, are uncommon hosts and highly resistant to WNV infection [22]. Therefore, chickens are not ideal to describe the avian immune response to WNV infection. Passeriformes and Galliformes are also highly divergent bird lineages, with distinctive immune gene repertoires and architecture [23].

As passerine birds are the main hosts for WNV, we have sought to develop a passerine model to study the impacts of WNV infection on a taxonomically appropriate host [24]. We have recently shown that zebra finches, *Taeniopygia guttata*, are moderately susceptible hosts for WNV [25]. That is, WNV rapidly disseminates to a variety of tissues and is detectable in most samples by 4 days post-inoculation (dpi). Despite rapid development of sufficient viremia for arthropod transmission, zebra finches develop anti-WNV antibodies, clear WNV by 14 dpi, and avoid significant mortality [25]. This moderate disease susceptibility is similar to what is observed in many natural WNV hosts. Zebra finches are also an established biomedical model system with a suite of genetic and genomic tools available [26].

In this study, we experimentally infected zebra finches and performed RNAseq to describe their transcriptional response up to the point of peak viremia. In doing so, we characterize the zebra finch immune response to WNV infection, explore expression of the avian RLR pathway in response to WNV, gain insights into the avian immune response to this widespread infectious disease, and uncover conserved evolutionary responses in avian and mammalian systems.

## 2. Results

### 2.1. Experimental infection

We challenged six individuals with 10^5^ plaque-forming units (PFU) WNV and sequenced RNA (Illumina RNAseq) isolated from spleens, an organ critical to the avian immune response. Three birds served as procedural controls and on day 0 were injected subcutaneously with 100 µl of BA1 media, as previously described [27]. Peak viremia occurs at 4.6 ± 1.7 dpi as quantified via RT-PCR [25] and thus, we characterized the transcriptional response leading to (2 dpi, *n* = 3) and at peak viral load (4 dpi, *n* = 3) in the present study. WNV RNA was detected by culture in lung and kidney RNA pools of two out of three birds sampled at day 2, and all three birds sampled at 4 dpi. These findings were verified by semi-quantitative RT-PCR. WNV is rarely detected in spleen by 2 dpi, but all birds previously inoculated at 10^5^ PFU developed WNV antibodies [25]. We therefore treated all six birds inoculated with WNV as being infected.

### 2.2. Sequencing results and read mapping

We obtained 18–30 million paired-end, 100 bp reads for each sample and removed 0.57–1.24% of the total bases after adapter trimming (electronic supplementary material, table S1). On average, 79.0–80.8% total trimmed reads mapped to the zebra finch reference genome (electronic supplementary material, table S2), corresponding to 18 618 Ensembl-annotated genes [28]. Of these, 14 114 genes averaged at least five mapped reads across all samples and were used for differential expression (DE) analyses.

### 2.3. Sample clustering and differential expression

We tested for DE in two ways: as pairwise comparisons between treatments with *DEseq2* [29] and as a time-course grouping genes into expression paths with *EBSeqHMM* [30]. To visualize patterns of expression variation among samples, we conducted principal component analysis (PCA) and distance-based clustering (electronic supplementary material, figures S1 and S2). The first three principal components explained 93.04% of the variance in gene expression, but none of the PCs were significantly correlated with treatment (ANOVA, PC1: *p* = 0.288, PC2: *p* = 0.956, PC3: *p* = 0.202).

**Figure 1.**
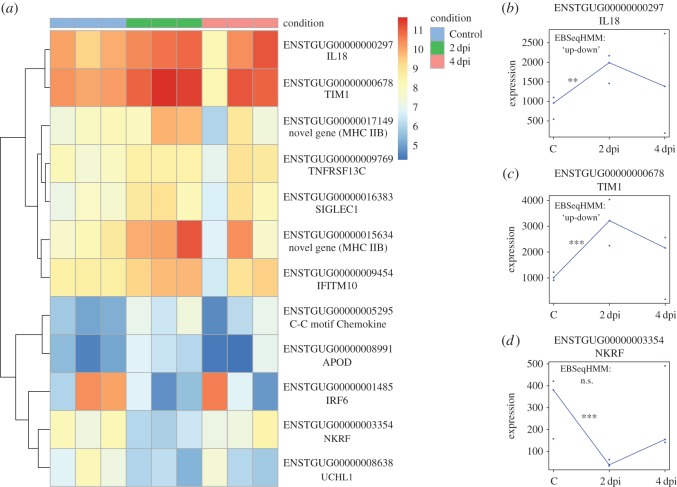
Immune genes differentially expressed between day 2 post-inoculation and Control. (*a*) Heatmap of expression levels (log transformed read counts) across all treatments of immune genes differentially expressed at 2 dpi relative to Control. (*b*–*d*) Expression values (normalized read counts) for three key immune genes and their regulation pattern classification by *EBSeqHMM*. Asterisks represent statistical significance in *DEseq2* analysis after FDR correction (*p < 0.10, **p < 0.05, ***p < 0.01).

**Figure 2.**
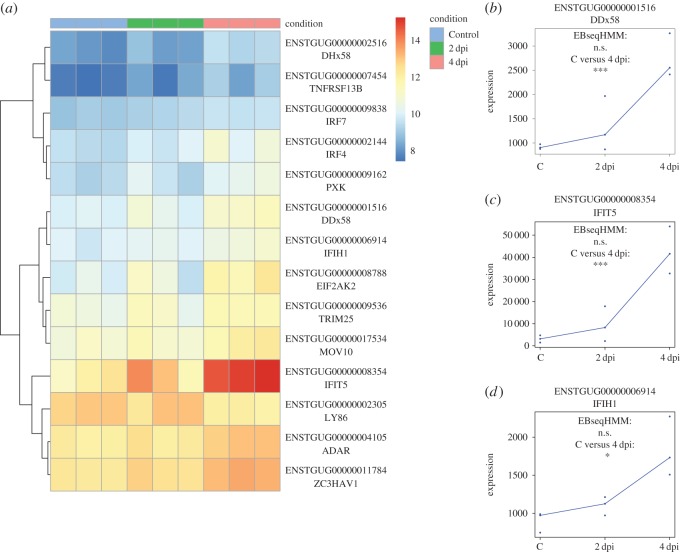
Immune genes differentially expressed between day 4 post-inoculation and Control. (*a*) Heatmap of expression levels (log transformed read counts) across all treatments of immune genes differentially expressed at 4 dpi relative to Control. (*b*–*d*) Expression values (normalized read counts) for three key immune genes and their regulation pattern classification by *EBSeqHMM*. Asterisks represent statistical significance in *DEseq2* analysis after FDR correction (*p < 0.10, **p < 0.05, ***p < 0.01).

**Figure 3.**
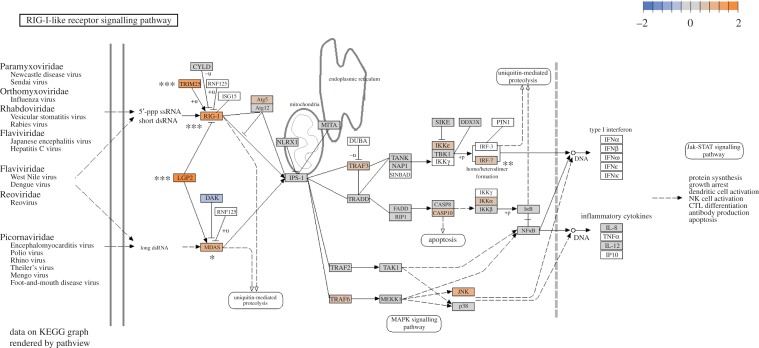
Regulation of the zebra finch RLR pathway. Colour represents log_2_ fold change between Control and 4 dpi. Asterisks represent statistical significance in *DEseq2* analysis after FDR correction (*p < 0.10, **p < 0.05, ***p < 0.01).

Although clustering analyses suggest that across the genome, much of the variation in expression was independent of the experimental treatment, pairwise comparisons revealed many genes that were regulated in response to infection (electronic supplementary material, table S3). When comparing Control versus 2 dpi, we found 161 differentially expressed genes (false discovery rate (FDR) < 0.10, average log_2_ fold-change (FC) = 1.74). This gene list includes several immune-related genes associated with the innate (e.g. IL18) and adaptive (e.g. MHC IIB) immune system (table 1, figure 1). Sixty-five genes were differentially expressed between Control and 4 dpi (average log_2_FC = 1.61), also with several immune-relevant genes including five genes in the RLR pathway (table 1, figures 2 and 3). Lastly, we observed 44 DE genes between 2 dpi versus 4 dpi individuals (average log_2_FC = 1.56). Three of these have described functions in immunity. We also combined 2 dpi and 4 dpi cohorts and compared with Control, but due to high variation in gene expression between days 2 and 4 dpi, we only found 16 DE genes (average log_2_FC = 1.64) between Control and Infected cohorts, one of which was associated with immunity.

**Table 1.** Candidate immune genes differentially expressed in the present study and comparisons with mammals.

Ensembl ID	gene name	log_2_ fold change	FDR	regulation pattern observed	regulation pattern in mammals	references
*Control versus Infected*						
ENSTGUG00000013615	NFKBIZ	0.73	0.064	up	up	[31]
*Control versus 2 dpi*						
ENSTGUG00000000297	IL18	1.01	0.010	up	no change	[32]
ENSTGUG00000000678	TIM1	1.49	7.99 × 10^−05^	up	up	[33,34]
ENSTGUG00000001485	IRF6	−2.09	0.037	down	up	[35]
ENSTGUG00000003354	NKRF	−2.35	4.85 × 10^−05^	down	unknown	
ENSTGUG00000005295	C-C motif chemokine	2.08	0.007	up	up	[31,35,36]
ENSTGUG00000008638	UCHL1	−1.91	0.029	down	unknown	
ENSTGUG00000008991	APOD	1.76	0.053	up	up	[37]
ENSTGUG00000009454	IFITM10	1.24	2.71 × 10^−04^	up	unknown	
ENSTGUG00000009769	TNFRSF13C	0.89	0.010	up	unknown	
ENSTGUG00000015634	Novel gene (MHC IIB)	2.23	0.001	up	up	[38]
ENSTGUG00000016383	SIGLEC1	1.39	0.046	up	up	[31]
ENSTGUG00000017149	Novel gene (MHC IIB)	1.57	0.099	up	up	[38]
*Control versus 4 dpi*						
ENSTGUG00000001516	DDx58	1.50	1.45 × 10^−08^	up	up	[31]
ENSTGUG00000002144	IRF4	1.39	0.022	up	up	[35]
ENSTGUG00000002305	LY86	−1.07	1.70 × 10^−05^	down	unknown	
ENSTGUG00000002516	DHx58	1.67	4.05 × 10^−06^	up	up	[31]
ENSTGUG00000004105	ADAR	1.13	1.70 × 10^−05^	up	up	[39]
ENSTGUG00000006914	IFIH1	0.95	0.093	up	up	[31]
ENSTGUG00000007454	TNFRSF13B	1.61	0.010	up	unknown	
ENSTGUG00000008354	IFIT5	2.97	1.07 × 10^−09^	up	unknown	
ENSTGUG00000008788	EIF2AK2	2.15	9.86 × 10^−07^	up	up	[35,40]
ENSTGUG00000009162	PXK	1.19	0.011	up	unknown	
ENSTGUG00000009536	TRIM25	1.24	0.001	up	up	[31]
ENSTGUG00000009838	IRF7	0.80	0.047	up	up	[31]
ENSTGUG00000011784	ZC3HAV1	1.31	7.09 × 10^−08^	up	up	[31]
ENSTGUG00000017534	MOV10	1.29	0.017	up	up	[39]
*2 dpi versus 4 dpi*						
ENSTGUG00000003354	NKRF	1.70	0.033	up	unknown	
ENSTGUG00000005206	ADA	−1.48	0.001	down	unknown	
ENSTGUG00000011784	ZC3HAV1	0.82	0.058	up	up	[31]

When analysed for DE as a time course in *EBSeqHMM*, 686 genes showed evidence of differential expression (posterior probability >0.99, FDR < 0.01). Most DE genes (*n* = 561) were suppressed relative to Controls on days 2 and 4 post-infection (‘Down-Down’). Seventy-five genes were ‘Up-Down’, 49 were ‘Down-Up’ and one was ‘Up-Up’. As expected, we found overlap of several immune genes between the two analyses. For example, IL18, APOD and IFITM10 are ‘Up-Down’ and this trend is reflected in the *DEseq2* Control versus 2 dpi analysis (figure 1).

### 2.4. Functional annotation of differentially expressed genes

To place differentially expressed genes into groups based on their biological function, we performed a gene ontology (GO) analysis using the *GOrilla* tool [41,42]. *GOrilla* uses the rank ordered of genes from *DEseq2* based on FDR-adjusted *p*-values. An enrichment score is calculated based on the number of genes in the top of the list that belong to a particular GO category relative to the expected number based on the frequency of functionally related genes in the total list. As above, we conducted GO analyses based on multiple pairwise analyses of gene expression. We found five significantly enriched GO categories between Control versus Infected (2 and 4 dpi) cohorts, of which ‘response to virus’ is the most significant (FDR = 0.008, Enrichment = 5.34) (table 2). We observed the strongest evidence of functional enrichment in the Control versus 2 dpi (*n* = 120) and Control versus 4 dpi (*n* = 36) contrasts (FDR < 0.05) (electronic supplementary material, table S4). Many enriched GO terms in the Control versus 2 dpi contrast are involved in membrane components, metabolism and cellular processes. Four GO categories were immune relevant, including ‘inflammatory response’ and ‘positive regulation of cytokine biosynthetic process’ (electronic supplementary material, table S4). The immune response manifests itself most strongly in the Control versus 4 dpi contrast with many enriched GO terms being immune-related categories (table 2; electronic supplementary material, table S4) and a broad range of differentially expressed immune genes (*n* = 14, table 1). Only two GO categories are enriched between 2 and 4 dpi: ‘inner mitochondrial membrane protein complex’ and ‘mitochondrial protein complex’ (table 2; electronic supplementary material, table S4).

**Table 2.** Top five statistically enriched gene ontology (GO) categories, FDR-adjusted *p*-value, and *GOrilla* enrichment score, among *DEseq2* pairwise comparisons. Enrichment is calculated as (b/n)/(B/N), where N is the total number of genes, B is the total number of genes associated with a specific GO term, n is the number of genes in the top of the input list, and b is the number of GO-term-associated genes in the top of the list [41,42].

GO ID	description	FDR	enrichment
*Control versus WNV*			
GO:0009615	response to virus	0.008	5.34
GO:0051276	chromosome organization	0.019	1.97
GO:1903047	mitotic cell cycle process	0.035	1.69
GO:0034723	DNA replication-dependent nucleosome assembly	0.039	14.27
GO:0006335	DNA replication-dependent nucleosome organization	0.048	14.27
*Control versus 2 dpi*			
GO:0044425	membrane part	1.77 × 10^−07^	1.29
GO:0098800	inner mitochondrial membrane protein complex	1.15 × 10^−06^	3.73
GO:0044459	plasma membrane part	2.66 × 10^−06^	1.98
GO:0031224	intrinsic component of membrane	4.04 × 10^−06^	1.35
GO:0098798	mitochondrial protein complex	4.06 × 10^−06^	3.32
*Control versus 4 dpi*			
GO:0009615	response to virus	0.001	19.23
GO:0051607	defence response to virus	0.002	51.27
GO:0060337	type I interferon signalling pathway	0.002	24.25
GO:0051707	response to other organism	0.002	11.73
GO:0098586	cellular response to virus	0.003	70.99
*2 dpi versus 4 dpi*			
GO:0098800	inner mitochondrial membrane protein complex	0.01	3.09
GO:0098798	mitochondrial protein complex	0.04	2.69

We also conducted a similar analysis of genes identified as DE by *EBseqHMM*, which revealed one (Up-Up), 199 (Up-Down), 69 (Down-Up) and 527 (Down-Down) significantly enriched GO categories (FDR < 0.05) (electronic supplementary material, table S5). Interestingly, Up-Down GO categories had the strongest representation of immune-related GO terms, including ‘immune response’ (FDR = 4.85 × 10^−4^) and ‘negative regulation of immune system process’ (FDR = 6.01 × 10^−4^). Among Down-Down genes, we observed enrichment of many metabolic and membrane processes and only one immune-related category (‘positive regulation of innate immune response’, FDR = 0.01). We found enrichment of mitochondrial components and processes among Down-Up genes, similar to the 2 dpi versus 4 dpi contrast in the *DEseq2* analysis. Additionally, 10 categories involved in immunoglobulin processes were significantly enriched among ‘Down-Up’ genes, driven by the presence of the joining chain of multimeric IgA and IgM (JCHAIN) gene. Lastly, as in the *DEseq2*-based analysis, we also detected a strong enrichment signature of membrane proteins. Genes annotated as ‘plasma membrane part’ were highly enriched among those showing an Up-Down pattern (FDR = 1.61 × 10^−12^, electronic supplementary material, table S5). Combined, we found broad overlap in GO representation between the *EBseqHMM* and *DEseq2* approaches.

In addition to placing genes into broad systematic functions in the GO analysis, we were also interested in placing our gene expression results in the context of immune pathways of interest. The RLR antiviral pathway is critical to WNV clearance in mammals [12] and appears important in mounting an immune response to avian influenza in ducks [43–45]. Using *Pathview v1.8.0* [46], we find that WNV infection induces the RLR pathway. Five genes, including the two RLR genes, DDx58 and IFIH1, which encode the Rig-I and MDA5 viral detection molecules, are significantly upregulated (table 1, figure 2; electronic supplementary material, figure S3). We detect expression of 36/37 genes in the pathway, many of which are also upregulated, though not always significantly (figure 3).

## 3. Discussion

We have characterized the zebra finch transcriptional response to WNV infection. Overall, we find that as in mammalian systems, components of both the adaptive and innate immune pathways are activated following infection. While WNV is primarily an avian-specific infectious disease, most work describing the host immune response to infection has been performed in mammals. Despite genomic, physiological and evolutionary differences between birds and mammals, the host immune response shows broad similarity between taxa (table 1).

We were particularly interested in the role of the innate RLR pathway. This pathway mounts an antiviral innate immune response and is critical for WNV detection and clearance in mammals [12]. We have shown here that the RLR pathway in zebra finches is induced by WNV infection. Five genes in this pathway are significantly upregulated at 4 dpi (figure 3; electronic supplementary material, figure S3), including DDx58 and IFIH1 (figure 2*b*,*d*), which encode molecules that recognize WNV particles in mammals [47]. This results in a corresponding over-representation of genes in the interferon signalling and regulation GO categories (table 2; electronic supplementary material, table S4). While no studies have investigated the role of the RLR following WNV infection in birds, this pathway appears important for avian influenza clearance in ducks [43–45], Buggy Creek virus clearance in house sparrows [48], and probably for the broad avian antiviral immune response, including WNV. Interestingly, chickens (*Gallus gallus*), which are often used as sentinels for WNV, have lost the gene encoding the DDx58 RLR during their evolution [43] yet do not develop disease post WNV infection [22]. This suggests that chickens respond to WNV using a Rig-I independent mechanism and highlights the importance of future work targeting the evolution of avian innate immunity.

We observed other parallels with mammals as well (table 1). For example, T-cell immunoglobulin mucin receptor 1 (TIM1) is upregulated at 2 dpi in zebra finches (figure 1*a*,*c*). In human cell lines, expression of TIM1 promotes infection of WNV virus-like particles (VLPs) [33,34], suggesting that the upregulation of TIM1 seen in zebra finches may promote viral entry as well. Similarly, C-C motif chemokine (ENSTGUG00000005295) is upregulated in our study at 2 dpi and in previous human cell line and mouse experiments, suggesting a conserved role in chemokine production following WNV infection [31,35,36]. Apolipoprotein D (APOD), a gene typically involved in brain injury and potentially responding to the neurodegenerative nature of WNV, is upregulated in WNV-infected mice [37], as well as in our study. Two interferon-stimulated genes (ISGs), ADAR and MOV10 are both significantly upregulated at 4 dpi relative to Control. Schoggins *et al*. [39] showed ADAR expression to enhance WNV replication and MOV10 expression to have antiviral activity. While further testing of these genes is needed to validate their roles in avian WNV infection, they nonetheless offer insights into a broad range of conserved responses between mammals and birds.

Within the adaptive immune response, the role of the MHC in the host response to WNV is also particularly interesting. The MHC plays a key role in antigen processing and presentation. The MHC comprises two main gene families (Class I & II) and both are upregulated in mammals following WNV infection [15,16,38]. Similarly, two genes encoding MHC class IIB proteins are significantly upregulated in zebra finches at 2 dpi (figure 1). Unlike mammals, however, we found that MHC class I is not significantly DE in any comparison (e.g. C versus 4 dpi, log_2_FC = 0.001, FDR = 0.99). In mammals, upregulation of MHC class I may not be adaptive for the host, as upregulation may be a mechanism by which the virus evades Natural Killer (NK) cell detection by the innate immune system [15]. It has also been suggested that MHC upregulation is a by-product of flavivirus assembly [15]. Interestingly, at 2 dpi, interleukin-18 (IL18) is significantly upregulated (table 1, figure 1*a*,*b*). IL18 can enhance NK cell activity [49] and is potentially a mechanism by which the immune system can counteract WNV evasion strategies via NK cell activation, although further testing is needed to quantify NK cell activity in zebra finches to support this hypothesis.

Despite many similarities, several immune genes differentially expressed in our analyses have not been previously reported in the mammalian WNV literature or are expressed differently in zebra finches (table 1). For example, at 2 dpi, the proinflammatory cytokine IL18 was significantly upregulated in zebra finches (figure 1), contrasting a previous study in human cell lines, which showed no difference in IL18 expression following WNV infection [32]. Furthermore, interferon regulatory factor 6 (IRF6) was downregulated at 2 dpi, but upregulated in human macrophages following infection [35]. Another significantly downregulated gene at 2 dpi, ubiquitin carboxyl-terminal hydrolase L1 (UCHL1), has been previously associated with pattern recognition receptor (PRR) pathway (e.g. RLR) function in human cell lines infected with high-risk human papilloma virus [50]. When upregulated, UCHL1 supresses PRR expression leading to viral evasion of the host immune response. However, downregulation of UCHL1 restores functional PRR pathways [50]. Thus, the downregulation of UCHL1 2 dpi in zebra finches may be associated with the upregulation of the PRR RLR pathway in this study (table 1, figure 3). Interferon-induced protein with tetratricopeptide repeats (IFIT) and interferon-inducible transmembrane protein (IFITM) gene families are known innate antiviral proteins and have been shown to restrict WNV entry in human cells lines [39,51]. Both IFIT5 and IFITM10 are upregulated (figure 2*a*,*c*) in our study and yet, to our knowledge, neither have previously been implicated in the WNV immune response. This potentially reveals an avian-specific function of IFIT5 and IFITM10. Lastly, several genes involved in metabolic and mitochondrial processes were DE in our analyses. Viral alteration of host metabolism typically benefits viral replication [52,53] and highlights the need for future work investigating the role of WNV on host physiology.

Functional enrichment of immune-related GO terms primarily appears in Up-Down path defined by *EBseqHMM* (electronic supplementary material, table S5), as many genes in the immune system are upregulated post-infection (table 1, figures 1 and 2). In both the *EBseqHMM* and *DEseq2* analyses, most of the significant immune GO categories are innate immune responses, although adaptive immune categories involved in immunoglobulin complexes and B & T cell proliferation appear in the EBseqHMM analysis (table 2; electronic supplementary material, tables S4 and S5). Similar to the mammalian model, broad organismal processes, encompassing both innate and adaptive immunity, are represented in the zebra finch response to WNV.

Like many passerine birds infected in nature, zebra finches are moderately susceptible to WNV, developing sufficient viremia to serve as competent hosts, but generally resist mortality due to infection [25]. While there are clear differences among treatments in terms of differentially expressed genes (table 1), the modest effect of treatment on overall expression profile (electronic supplementary material, figures S1 and S2) may be a reflection of this moderate susceptibility. Most zebra finches are able to clear WNV infection by 14 dpi [25]. WNV infection intensity varies among tissues [20], but due to the spleen's important role in the avian immune system [54,55] we expect the results presented here to be representative of the overall immune response. Although we expect to have missed some genes that are regulated in response to infection, *DEseq2* has been shown to perform very well (low false positive rate) in experiments with a sample size of three [56]. Further studies will also be required to document more subtle, and tissue-specific patterns of gene regulation in response to infection. We note that we only sampled our control group at 4 dpi and thus, do not have a direct procedural control at 2 dpi. Changes in gene expression at 2 dpi therefore could be in part due to the injection itself. Pronounced DE of immune-related genes at 2 dpi, however, suggests that changes in gene expression were driven by WNV infection rather than by the injection, which might be predicted to trigger a more general stress response.

We have begun to develop the zebra finch as an avian model for the host response to WNV infection. We show here that in terms of gene expression, the zebra finch immune response is largely conserved with that seen in mammalian-based studies (table 1). Additionally, we identify many components of the immune system that have not been previously implicated in the host immune response to WNV. This potentially reveals an avian-specific immune response and highlights avenues for future research. Combined with our recent immunological characterization [25], we have broadly described the immune response of a moderately susceptible avian host for WNV. This sets the stage for future comparative work to uncover the genetic basis of variable avian susceptibility to WNV infection.

## 4. Methods

### 4.1. Experimental set-up

All animal use was approved by the USGS National Wildlife Health Center Institutional Animal Care and Use Committee (IACUC Protocol: EP120521) and this study was performed in accordance with USGS IACUC guidelines. The experimental infection set-up is described in detail in [25]. Briefly, nine female zebra finches were randomly divided into three cohorts, one unchallenged and two challenged (*n* = 3 each). Birds were challenged subcutaneously with 100 µl BA1 media containing 10^5^ plaque-forming units (PFU) of the 1999 American crow isolate of WNV (NWHC 16399-3) and sacrificed at 2 and 4 dpi, corresponding to peak viremia. Uninfected individuals were injected with 100 µl BA1 media and sacrificed at 4 dpi. WNV infection was confirmed by RT-PCR, as previously described [26], in lung and kidney pooled tissue [25]. Due to the critical role of the spleen in the initiation of the immune response, and its common use in experimental infection gene expression studies [57–60], we focused our study on gene expression in the spleen. Spleens from each individual were removed, placed into RNAlater (Qiagen, Valencia, CA, USA), and frozen at −80°C until RNA extraction.

### 4.2. RNA extraction and sequencing

Whole spleen tissue was homogenized in Tri-Reagent (Molecular Research Company) and total RNA was purified with a Qiagen RNeasy (Valencia, CA, USA) mini kit following the manufacturer's protocol. RNA was DNAse treated and purified. Purified RNA was quality assessed on a Bioanalyzer (Agilent, Wilmington, DE, USA) to ensure RNA quality before sequencing (RIN = 6.6–8.1). All library prep and sequencing was performed at the University of Illinois Roy J. Carver Biotechnology Center. A library for each sample was prepared with an Illumina TruSeq Stranded RNA sample prep kit. All libraries were pooled, quantitated by qPCR, and sequenced on one lane of an Illumina HiSeq 2000 with a TruSeq SBS Sequencing Kit producing paired-end 100 nt reads. Reads were analysed with Casava 1.8.2 following manufacturer's instructions (Illumina, San Diego, CA). Sequencing data from this study have been deposited in the NCBI Sequence Read Archive (BioProject: PRJNA352507).

### 4.3. Adapter trimming and read mapping

We removed Illumina adapters from reads with *Trim Galore! v0.3.7* (http://www.bioinformatics.babraham.ac.uk/projects/trim_galore/) which makes use of *Cutadapt v1.7.1* [61]. Reads were then mapped to the zebra finch genome (*v3.2.74*,26) using *TopHat v2.0.13* [62], which uses the aligner *Bowtie v2.2.4* [63]. We specified the library type as fr-firststrand in *TopHat2*. Successfully mapped reads were converted from SAM to BAM format with *SAMtools View v1.2* [64,65] and counted in *htseq-count v0.6.0* specifying ‘-s rev’ [66]. This assigned zebra finch Ensembl gene IDs and we only retained genes with >5X mapping across each sample.

### 4.4. Differential expression

Gene counts were then normalized for read-depth and analysed for DE in *DEseq2 v1.8.1* [29]. We analysed DE across four comparisons: Control versus Infected, Control versus 2 dpi, Control versus 4 dpi, and 2 dpi versus 4 dpi. We visualized expression profiles in *R v.3.3.0* [67] by PCA with the R package pcaExplorer [68], and hierarchical clustering heat maps with the ggplot2 library [69] following the *DEseq2* manual. *DEseq2* tests for DE with a Wald test and genes were considered differentially expressed if the Benjamini & Hochberg [70] FDR correction for multiple testing *p*-value less than 0.10. We chose this significance threshold as *DEseq2* is generally conservative in classifying DE [71]. Furthermore, this cut-off is used by the *DEseq2* authors [29] and has been used in other RNAseq experimental infection studies [72]. We plotted genes of interest individually with the plotCounts function in *DEseq2* and clustered expression profiles of these genes with the pheatmap R library to view expression levels across samples and treatments.

We tested DE genes for enriched gene ontology (GO) categories with *GOrilla* [41,42]. *GOrilla* does not perform analyses with zebra finch Ensembl IDs, so we converted zebra finch Ensembl IDs to human Ensembl IDs using BioMart [73]. We used this set of 10 152 genes for analysis. For each pairwise comparison, we used the FDR ranked order DE genes from *DEseq2*. Statistical significance was determined with *p*-values corrected for multiple hypothesis testing (*p* < 0.05) using the Benjamini & Hochberg method [70]. To visualize DE results in the context of the RLR pathway, we used *Pathview v1.8.0* [46] to plot the log fold change of each gene detected in our dataset into the Kyoto Encyclopedia of Genes and Genomes (KEGG) pathway (KEGG ID = 04622) [74,75].

### 4.5. Time-course gene expression

In addition to the pair-wise comparisons performed in *DEseq2*, we were interested in understanding how clusters of genes are differentially expressed over the time course of infection. Thus, we performed DE analyses in *EBSeqHMM* [30]. *EBSeqHMM* uses a Bayesian approach with a hidden Markov model to identify DE between ordered conditions. Genes are then grouped into expression paths (i.e. ‘Up-Down’, ‘Down-Down’), in which DE occurs when expression paths change between at least one adjacent condition. For example, a gene upregulated at both 2 dpi relative to Control and 4 dpi relative to 2 dpi would be classified as ‘Up-Up’. We included three time points, with Control individuals classified as t1, 2 dpi as t2 and 4 dpi as t3. Genes were considered DE at posterior probability >0.99 and FDR <0.01. We chose a more stringent cut-off in this analysis as *EBseq* can be liberal in classifying differential expression [71] and based on visual inspection of expression profiles. We ordered genes based on posterior-probability for each expression path and performed the GO analysis described above.

## Supplementary Material

Supplemental Information

## Supplementary Material

Supplemental Table S3

## Supplementary Material

Supplemental Table S4

## Supplementary Material

Supplemental Table S5

## Data accessibility

Sequencing data have been deposited in the Sequence Read Archive (SRA) under accession PRJNA352507.

## References

[1] Chancey C, Grinev A, Volkova E, Rios M 2015 The global ecology and epidemiology of West Nile virus. Biomed Res. Int. 2015, 20 (doi:10.1155/2015/376230) 10.1155/2015/376230 PMC4383390 25866777

[2] Pérez-Ramírez E, Llorente F, Jiménez-Clavero MÁ 2014 Experimental infections of wild birds with West Nile virus. Viruses 6, 752–781. (doi:10.3390/v6020752) 24531334 10.3390/v6020752 PMC3939481

[3] McLean RG, Ubico SR, Docherty DE, Hansen WR, Sileo L, McNamara TS 2001 West Nile virus transmission and ecology in birds. Ann. N. Y. Acad. Sci. 951, 54–57. (doi:10.1111/j.1749-6632.2001.tb02684.x) 11797804 10.1111/j.1749-6632.2001.tb02684.x

[4] LaDeau SL, Kilpatrick a M, Marra PP 2007 West Nile virus emergence and large-scale declines of North American bird populations. Nature 447, 710–713. (doi:10.1038/nature05829) 17507930 10.1038/nature05829

[5] George TL, Harrigan RJ, LaManna JA, DeSante DF, Saracco JF, Smith TB 2015 Persistent impacts of West Nile virus on North American bird populations. Proc. Natl Acad. Sci. USA 112, 14 290–14 294. (doi:10.1073/pnas.1507747112) 26578774 10.1073/pnas.1507747112 PMC4655513

[6] Kilpatrick AM, Daszak P, Jones MJ, Marra PP, Kramer LD 2006 Host heterogeneity dominates West Nile virus transmission. Proc. R. Soc. B 273, 2327–2333. (doi:10.1098/rspb.2006.3575) 10.1098/rspb.2006.3575 PMC1636093 16928635

[7] Komar N, Langevin S, Hinten S, Nemeth N, Edwards E, Hettler D, Davis B, Bowen R, Bunning M 2003 Experimental infection of North American birds with the New York 1999 strain of West Nile virus. Emerg. Infect. Dis. 9, 311–322. (doi:10.3201/eid0903.020628) 12643825 10.3201/eid0903.020628 PMC2958552

[8] Suthar MS, Diamond MS, Gale M 2013 West Nile virus infection and immunity. Nat. Rev. Microbiol. 11, 115–128. (doi:10.1038/nrmicro2950) 23321534 10.1038/nrmicro2950

[9] Diamond MS, Shrestha B, Mehlhop E, Sitati E, Engle M 2003 Innate and adaptive immune responses determine protection against disseminated infection by West Nile encephalitis virus. Viral Immunol. 16, 259–278. (doi:10.1089/088282403322396082) 14583143 10.1089/088282403322396082

[10] Diamond M, Shrestha B, Marri A 2003 B cells and antibody play critical roles in the immediate defense of disseminated infection by West Nile encephalitis virus. J. Virol. 77, 2578–2586. (doi:10.1128/JVI.77.4.2578) 12551996 10.1128/JVI.77.4.2578-2586.2003 PMC141119

[11] Diamond MS, Gale M 2012 Cell-intrinsic innate immune control of West Nile virus infection. Trends Immunol. 33, 522–530. (doi:10.1016/j.it.2012.05.008) 22726607 10.1016/j.it.2012.05.008 PMC3461102

[12] Errett JS, Suthar MS, McMillan A, Diamond MS, Gale M 2013 The essential, nonredundant roles of RIG-I and MDA5 in detecting and controlling West Nile virus infection. J. Virol. 87, 11 416–11 425. (doi:10.1128/JVI.01488-13) 10.1128/JVI.01488-13 PMC3807316 23966395

[13] Sitati EM, Diamond MS 2006 CD4+ T-cell responses are required for clearance of West Nile virus from the central nervous system. J. Virol. 80, 12 060–12 069. (doi:10.1128/JVI.01650-06) 10.1128/JVI.01650-06 PMC1676257 17035323

[14] Shrestha B, Diamond M 2004 Role of CD8+ T cells in control of West Nile virus infection. J. Virol. 78, 8312–8321. (doi:10.1128/JVI.78.15.8312) 15254203 10.1128/JVI.78.15.8312-8321.2004 PMC446114

[15] Lobigs M, Müllbacher A, Regner M 2003 MHC class I up-regulation by flaviviruses: immune interaction with unknown advantage to host or pathogen. Immunol. Cell Biol. 81, 217–223. (doi:10.1046/j.1440-1711.2003.01161.x) 12752686 10.1046/j.1440-1711.2003.01161.x

[16] Cheng Y, King NJC, Kesson AM 2004 Major histocompatibility complex class I (MHC-I) induction by West Nile virus: involvement of 2 signaling pathways in MHC-I up-regulation. J. Infect. Dis. 189, 658–668. (doi:10.1086/381501) 14767820 10.1086/381501

[17] Hewitt EW 2003 The MHC class I antigen presentation pathway: strategies for viral immune evasion. Immunology 110, 163–169. (doi:10.1046/j.1365-2567.2003.01738.x) 14511229 10.1046/j.1365-2567.2003.01738.x PMC1783040

[18] Petersen JL, Morris CR, Solheim JC 2003 Virus evasion of MHC class I molecule presentation. J. Immunol. 171, 4473–4478. (doi:10.4049/jimmunol.171.9.4473) 14568919 10.4049/jimmunol.171.9.4473

[19] Fair JM, Nemeth NM, Taylor-McCabe KJ, Shou Y, Marrone BL 2011 Clinical and acquired immunologic responses to West Nile virus infection of domestic chickens (*Gallus gallus domesticus*). Poult. Sci. 90, 328–336. (doi:10.3382/ps.2010-00809) 21248329 10.3382/ps.2010-00809

[20] Gamino V, Höfle U 2013 Pathology and tissue tropism of natural West Nile virus infection in birds: a review. Vet. Res. 44, 39 (doi:10.1186/1297-9716-44-39) 23731695 10.1186/1297-9716-44-39 PMC3686667

[21] Tag-El-Din-Hassan HT, Sasaki N, Moritoh K, Torigoe D, Maeda A, Agui T 2012 The chicken 2′-5′ oligoadenylate synthetase A inhibits the replication of West Nile virus. Jpn. J. Vet. Res. 60, 95–103. 23094584

[22] Langevin SA, Bunning M, Davis B, Komar N 2001 Experimental infection of chickens as candidate sentinels for West Nile virus. Emerg. Infect. Dis. 7, 726–729. (doi:10.3201/eid0704.017422) 11585538 10.3201/eid0704.010422 PMC2631771

[23] Balakrishnan CN et al. 2010 Gene duplication and fragmentation in the zebra finch major histocompatibility complex. BMC Biol. 8, 29 (doi:10.1186/1741-7007-8-29) 20359332 10.1186/1741-7007-8-29 PMC2907588

[24] Bean AGD, Baker ML, Stewart CR, Cowled C, Deffrasnes C, Wang L-F, Lowenthal JW 2013 Studying immunity to zoonotic diseases in the natural host—keeping it real. Nat. Rev. Immunol. 13, 851–861. (doi:10.1038/nri3551) 24157573 10.1038/nri3551 PMC7098194

[25] Hofmeister EK, Lund M, Shearn-Bochsler V, Balakrishnan CN 2017 Susceptibility and antibody response of the laboratory model zebra finch (*Taeniopygia guttata*) to West Nile virus. PLoS ONE 12, e0167876 (doi:10.1371/journal.pone.0167876) 28045891 10.1371/journal.pone.0167876 PMC5207765

[26] Warren WC et al. 2010 The genome of a songbird. Nature 464, 757–762. (doi:10.1038/nature08819) 20360741 10.1038/nature08819 PMC3187626

[27] Hofmeister EK, Dusek RJ, Fassbinder-Orth C, Owen B, Franson JC 2016 Susceptibility and antibody response of vesper sparrows (*Pooecetes gramineus*) to West Nile virus: a potential amplification host in sagebrush-grassland habitat. J. Wildl. Dis. 52, 345–353. (doi:10.7589/2015-06-148) 26981692 10.7589/2015-06-148

[28] Yates A et al. 2016 Ensembl 2016. Nucleic Acids Res. 44, D710–D716. (doi:10.1093/nar/gkv1157) 26687719 10.1093/nar/gkv1157 PMC4702834

[29] Love MI, Huber W, Anders S 2014 Moderated estimation of fold change and dispersion for RNA-seq data with DESeq2. Genome Biol. 15, 550 (doi:10.1186/s13059-014-0550-8) 25516281 10.1186/s13059-014-0550-8 PMC4302049

[30] Leng N et al. 2015 EBSeq-HMM: a Bayesian approach for identifying gene-expression changes in ordered RNA-seq experiments. Bioinformatics 31, 2614–2622. (doi:10.1093/bioinformatics/btv193) 25847007 10.1093/bioinformatics/btv193 PMC4528625

[31] Kumar M, Belcaid M, Nerurkar VR 2016 Identification of host genes leading to West Nile virus encephalitis in mice brain using RNA-seq analysis. Sci. Rep. 6, 26350 (doi:10.1038/srep26350) 27211830 10.1038/srep26350 PMC4876452

[32] Kumar M, Verma S, Nerurkar VR 2010 Pro-inflammatory cytokines derived from West Nile virus (WNV)-infected SK-N-SH cells mediate neuroinflammatory markers and neuronal death. J. Neuroinflammation 7, 73 (doi:10.1186/1742-2094-7-73) 21034511 10.1186/1742-2094-7-73 PMC2984415

[33] Jemielity S et al. 2013 TIM-family proteins promote infection of multiple enveloped viruses through virion-associated phosphatidylserine. PLoS Pathog. 9, e1003232 (doi:10.1371/journal.ppat.1003232) 23555248 10.1371/journal.ppat.1003232 PMC3610696

[34] Amara A, Mercer J 2015 Viral apoptotic mimicry. Nat. Rev. Microbiol. 13, 461–469. (doi:10.1038/nrmicro3469) 26052667 10.1038/nrmicro3469 PMC7097103

[35] Qian F et al. 2013 Identification of genes critical for resistance to infection by West Nile virus using RNA-seq analysis. Viruses 5, 1664–1681, (doi:10.3390/v5071664) 23881275 10.3390/v5071664 PMC3738954

[36] Hussmann KL, Fredericksen BL 2014 Differential induction of CCL5 by pathogenic and non-pathogenic strains of West Nile virus in brain endothelial cells and astrocytes. J. Gen. Virol. 95, 862–867. (doi:10.1099/vir.0.060558-0) 24413421 10.1099/vir.0.060558-0 PMC3973477

[37] Venter M, Myers TG, Wilson MA, Kindt TJ, Paweska JT, Burt FJ, Leman PA, Swanepoel R 2005 Gene expression in mice infected with West Nile virus strains of different neurovirulence. Virology 342, 119–140. (doi:10.1016/j.virol.2005.07.013) 16125213 10.1016/j.virol.2005.07.013

[38] Liu Y, King N, Kesson A, Blanden RV, Müllbacher A 1989 Flavivirus infection up-regulates the expression of class I and class II major histocompatibility antigens on and enhances T cell recognition of astrocytes *in vitro*. J. Neuroimmunol. 21, 157–168. (doi:10.1016/0165-5728(89)90171-9) 2463998 10.1016/0165-5728(89)90171-9 PMC7119853

[39] Schoggins JJW, Wilson SJS, Panis M, Murphy MMY, Jones CCT, Bieniasz P, Rice CM 2011 A diverse array of gene products are effectors of the type I interferon antiviral response. Nature 472, 481–485. (doi:10.1038/nature09907) 21478870 10.1038/nature09907 PMC3409588

[40] Elbahesh H, Scherbik S V., Brinton MA 2011 West Nile virus infection does not induce PKR activation in rodent cells. Virology 421, 51–60. (doi:10.1016/j.virol.2011.08.008) 21982595 10.1016/j.virol.2011.08.008 PMC3208726

[41] Eden E, Lipson D, Yogev S, Yakhini Z 2007 Discovering motifs in ranked lists of DNA sequences. PLoS Comput. Biol. 3, e39 (doi:10.1371/journal.pcbi.0030039) 17381235 10.1371/journal.pcbi.0030039 PMC1829477

[42] Eden E, Navon R, Steinfeld I, Lipson D, Yakhini Z 2009 GOrilla: a tool for discovery and visualization of enriched GO terms in ranked gene lists. BMC Bioinformatics 10, 48 (doi:10.1186/1471-2105-10-48) 19192299 10.1186/1471-2105-10-48 PMC2644678

[43] Barber MR, Aldridge JR Jr, Webster RG, Magor KE 2010 Association of RIG-I with innate immunity of ducks to influenza. Proc. Natl Acad. Sci. USA 107, 5913–5918. 20308570 10.1073/pnas.1001755107 PMC2851864

[44] Huang Y et al. 2013 The duck genome and transcriptome provide insight into an avian influenza virus reservoir species. Nat. Genet. 45, 776–783. (doi:10.1038/ng.2657) 23749191 10.1038/ng.2657 PMC4003391

[45] Wei L, Cui J, Song Y, Zhang S, Han F, Yuan R, Gong L, Jiao P, Liao M 2014 Duck MDA5 functions in innate immunity against H5N1 highly pathogenic avian influenza virus infections. Vet. Res. 45, 66 (doi:10.1186/1297-9716-45-66; doi:10.1186/1297-9716-45-66) 24939427 10.1186/1297-9716-45-66 PMC4079828

[46] Luo W, Brouwer C 2013 Pathview: an R/Bioconductor package for pathway-based data integration and visualization. Bioinformatics 29, 1830–1831. (doi:10.1093/bioinformatics/btt285) 23740750 10.1093/bioinformatics/btt285 PMC3702256

[47] Takeuchi O, Akira S 2009 Innate immunity to virus infection. Immunol. Rev. 227, 75–86. (doi:10.1111/j.1600-065X.2008.00737.x) 19120477 10.1111/j.1600-065X.2008.00737.x PMC5489343

[48] Fassbinder-Orth CA, Barak VA, Rainwater EL, Altrichter AM 2014 Buggy Creek virus (Togaviridae: alphavirus) upregulates expression of pattern recognition receptors and interferons in house sparrows (*Passer domesticus*). Vector Borne Zoonotic Dis. 14, 439–446. (doi:10.1089/vbz.2013.1531) 24866749 10.1089/vbz.2013.1531 PMC4050711

[49] Watzl C 2014 How to trigger a killer: modulation of natural killer cell reactivity on many levels. Adv. Immunol. 124, 137–170. (doi:10.1016/B978-0-12-800147-9.00005-4) 25175775 10.1016/B978-0-12-800147-9.00005-4

[50] Karim R et al. 2013 Human papillomavirus (HPV) upregulates the cellular deubiquitinase UCHL1 to suppress the keratinocyte's innate immune response. PLoS Pathog. 9, e1003384 (doi:10.1371/journal.ppat.1003384) 23717208 10.1371/journal.ppat.1003384 PMC3662672

[51] Brass AL et al. 2009 The IFITM proteins mediate cellular resistance to Influenza A H1N1 virus, West Nile virus, and Dengue virus. Cell 139, 1243–1254. (doi:10.1016/j.cell.2009.12.017) 20064371 10.1016/j.cell.2009.12.017 PMC2824905

[52] El-Bacha T, Da Poian AT 2013 Virus-induced changes in mitochondrial bioenergetics as potential targets for therapy. Int. J. Biochem. Cell Biol. 45, 41–46. (doi:10.1016/j.biocel.2012.09.021) 23036789 10.1016/j.biocel.2012.09.021

[53] Zeidler DJ, Fernandes-Siqueira OL, Barbosa MG, Da Poian TA 2017 Non-canonical roles of Dengue virus non-structural proteins. Viruses 9, 42 (doi:10.3390/v9030042) 10.3390/v9030042 PMC5371797 28335410

[54] Jeurissen SH 1993 The role of various compartments in the chicken spleen during an antigen-specific humoral response. Immunology 80, 29–33. 8244460 PMC1422104

[55] Smith KG, Hunt JL 2004 On the use of spleen mass as a measure of avian immune system strength. Oecologia 138, 28–31. (doi:10.1007/s00442-003-1409-y) 14576931 10.1007/s00442-003-1409-y

[56] Schurch NJ et al. 2016 How many biological replicates are needed in an RNA-seq experiment and which differential expression tool should you use? RNA 22, 839–851. (doi:10.1261/rna.053959.115) 27022035 10.1261/rna.053959.115 PMC4878611

[57] Bonneaud C, Balenger SL, Russell AF, Zhang J, Hill GE, Edwards SV 2011 Rapid evolution of disease resistance is accompanied by functional changes in gene expression in a wild bird. Proc. Natl Acad. Sci. USA 108, 7866–7871. (doi:10.1073/pnas.1018580108) 21525409 10.1073/pnas.1018580108 PMC3093480

[58] Zhang Q, Hill GE, Edwards SV, Backstrom N 2014 A house finch (*Haemorhous mexicanus*) spleen transcriptome reveals intra- and interspecific patterns of gene expression, alternative splicing and genetic diversity in passerines. BMC Genomics 15, 305 (doi:10.1186/1471-2164-15-305) 24758272 10.1186/1471-2164-15-305 PMC4235107

[59] Hamzić E et al. 2016 RNA sequencing-based analysis of the spleen transcriptome following infectious bronchitis virus infection of chickens selected for different mannose-binding lectin serum concentrations. BMC Genomics 17, 82 (doi:10.1186/s12864-016-2403-1) 26819139 10.1186/s12864-016-2403-1 PMC4729133

[60] Van Goor A, Ashwell CM, Persia ME, Rothschild MF, Schmidt CJ, Lamont SJ 2017 Unique genetic responses revealed in RNA-seq of the spleen of chickens stimulated with lipopolysaccharide and short-term heat. PLoS ONE 12, e0171414 (doi:10.1371/journal.pone.0171414) 28166270 10.1371/journal.pone.0171414 PMC5293231

[61] Martin M 2011 Cutadapt removes adapter sequences from high-throughput sequencing reads. EMBnet.journal 17, 10 (doi:10.14806/ej.17.1.200)

[62] Kim D, Pertea G, Trapnell C, Pimentel H, Kelley R, Salzberg SL 2013 Tophat2: accurate alignment of transcriptomes in the presence of insertions, deletions and gene fusions. Genome Biol. 14, R36 (doi:10.1186/gb-2013-14-4-r36) 23618408 10.1186/gb-2013-14-4-r36 PMC4053844

[63] Langmead B, Salzberg SL 2012 Fast gapped-read alignment with Bowtie 2. Nat. Methods 9, 357–359. (doi:10.1038/nmeth.1923) 22388286 10.1038/nmeth.1923 PMC3322381

[64] Li H 2011 A statistical framework for SNP calling, mutation discovery, association mapping and population genetical parameter estimation from sequencing data. Bioinformatics 27, 2987–2993. (doi:10.1093/bioinformatics/btr509) 21903627 10.1093/bioinformatics/btr509 PMC3198575

[65] Li H, Handsaker B, Wysoker A, Fennell T, Ruan J, Homer N, Marth G, Abecasis G, Durbin R 2009 The Sequence Alignment/Map format and SAMtools. Bioinformatics 25, 2078–2079. (doi:10.1093/bioinformatics/btp352) 19505943 10.1093/bioinformatics/btp352 PMC2723002

[66] Anders S, Pyl PT, Huber W 2015 HTSeq: a Python framework to work with high-throughput sequencing data. Bioinformatics 31, 166–169. (doi:10.1093/bioinformatics/btu638) 25260700 10.1093/bioinformatics/btu638 PMC4287950

[67] R Core Team. 2016 R: a language and environment for statistical computing. Vienna, Austria: R Foundation for Statistical Computing See https://www.R-project.org/.

[68] Marini F 2016 pcaExplorer: interactive visualization of RNA-seq data using a principal components approach. R package version 1.1.2, https://github.com/federicomarini/pcaExplorer

[69] Wickham H 2009 Ggplot2: elegant graphics for data analysis. New York, NY: Springer-Verlag.

[70] Benjamini Y, Hochberg Y 1995 Controlling the false discovery rate: a practical and powerful approach to multiple testing. J. R. Stat. Soc. 57, 289–300. (doi:10.2307/2346101)

[71] Seyednasrollah F, Laiho A, Elo LL 2015 Comparison of software packages for detecting differential expression in RNA-seq studies. Brief. Bioinform. 16, 59–70. (doi:10.1093/bib/bbt086) 24300110 10.1093/bib/bbt086 PMC4293378

[72] Videvall E, Cornwallis CK, Palinauskas V, Valkiūnas G, Hellgren O 2015 The avian transcriptome response to malaria infection. Mol. Biol. Evol. 32, 1255–1267. (doi:10.1093/molbev/msv016) 25636457 10.1093/molbev/msv016 PMC4408411

[73] Kinsella RJ et al. 2011 Ensembl BioMarts: a hub for data retrieval across taxonomic space. Database (Oxford) 2011, bar030 (doi:10.1093/database/bar030) 21785142 10.1093/database/bar030 PMC3170168

[74] Kanehisa M, Goto S 2000 KEGG: Kyoto Encyclopedia of Genes and Genomes. Nucleic Acids Res. 28, 27–30. (doi:10.1093/nar/28.1.27) 10592173 10.1093/nar/28.1.27 PMC102409

[75] Kanehisa M, Sato Y, Kawashima M, Furumichi M, Tanabe M 2016 KEGG as a reference resource for gene and protein annotation. Nucleic Acids Res. 44, D457–D462. (doi:10.1093/nar/gkv1070) 26476454 10.1093/nar/gkv1070 PMC4702792

