# Supplementary material for: Transcriptional response to West Nile virus infection in the zebra finch (Taeniopygia guttata)
Source: R Soc Open Sci. 2017 Jun 28;4(6):170296. doi: 10.1098/rsos.170296 (PMC5493925; doi:10.1098/rsos.170296)
Supplement: Supplemental Information [file rsos170296supp1.pdf]

Title: Transcriptional response to West Nile virus infection in the zebra finch (*Taeniopygia guttata*), a songbird model for immune function

Authors:

Daniel J Newhouse[1*], Erik K Hofmeister[2], Christopher N Balakrishnan[1]

[1] Howell Science Complex, East Carolina University, Greenville, North Carolina, 27858, USA

[2] U.S. Geological Survey, National Wildlife Health Center, 6006 Schroeder Road, Madison, Wisconsin 53711, USA

[*] Corresponding Author: newhoused12@students.ecu.edu

Supplemental Table S1. Number of reads and bases before and after adaptor trimming.

Sample	Raw Reads		Reads Trimmed		% Reads Trimmed		Raw Bases		Bases Trimmed		% Bases Trimmed	
	Read 1	Read 2	Read 1	Read 2	Read 1	Read 2	Read 1	Read 2	Read 1	Read 2	Read 1	Read 2
2dpi_1	23131286	23131286	7799698	8201136	33.7	35.5	2313128600	2313128600	13509100	14258319	0.58	0.62
Control_1	26984171	26984171	9759310	10199697	36.2	37.8	2698417100	2698417100	32908351	33473407	1.22	1.24
4dpi_1	24805127	24805127	8342723	8796081	33.6	35.5	2480512700	2480512700	14277592	15018376	0.58	0.61
4dpi_2	24457354	24457354	8330634	8697176	34.1	35.6	2445735400	2445735400	14588500	15300486	0.6	0.63
Control_2	18802029	18802029	6232427	6542397	33.1	34.8	1880202900	1880202900	10295134	10805314	0.55	0.57
Control_3	28830024	28830024	9909513	10275476	34.4	35.6	2883002400	2883002400	17480176	18157676	0.61	0.63
4dpi_3	29857919	29857919	10229865	10692817	34.3	35.8	2985791900	2985791900	18353596	19257146	0.61	0.64
2dpi_2	30778066	30778066	10879773	11327325	35.3	36.8	3077806600	3077806600	23349003	24199636	0.76	0.79
2dpi_3	24120394	24120394	8312658	8769098	34.5	36.4	2412039400	2412039400	16832733	17680667	0.7	0.73

Supplemental Table S2. Mapping rates of trimmed reads to the zebra finch reference genome with *TopHat2*

Sample	Mapping Rate:
2dpi_1	80.80%
Control_1	79.10%
4dpi_1	79.10%
4dpi_2	79.80%
Control_2	79.30%
Control_3	79.00%
4dpi_3	79.30%
2dpi_2	79.60%
2dpi_3	79.10%

Supplemental Table S3. Spreadsheet of differentially expressed genes (adjusted p value < 0.10) from the *DEseq2* analysis.

*File available for download in online supplement

Supplemental Table S4. Spreadsheet of GO results from *DEseq2* analysis.

*File available for download in online supplement

Supplemental Table S5. Spreadsheet of GO results from *EBseqHMM* analysis.

*File available for download in online supplement

Supplemental Figure S1. PCA of all individuals, colored by treatment, used in this study.

Supplemental Figure S2. Heatmap of all individuals used in this study.

Supplemental Figure S3. Normalized counts for the five RLR genes differentially expressed at 4dpi relative to Control

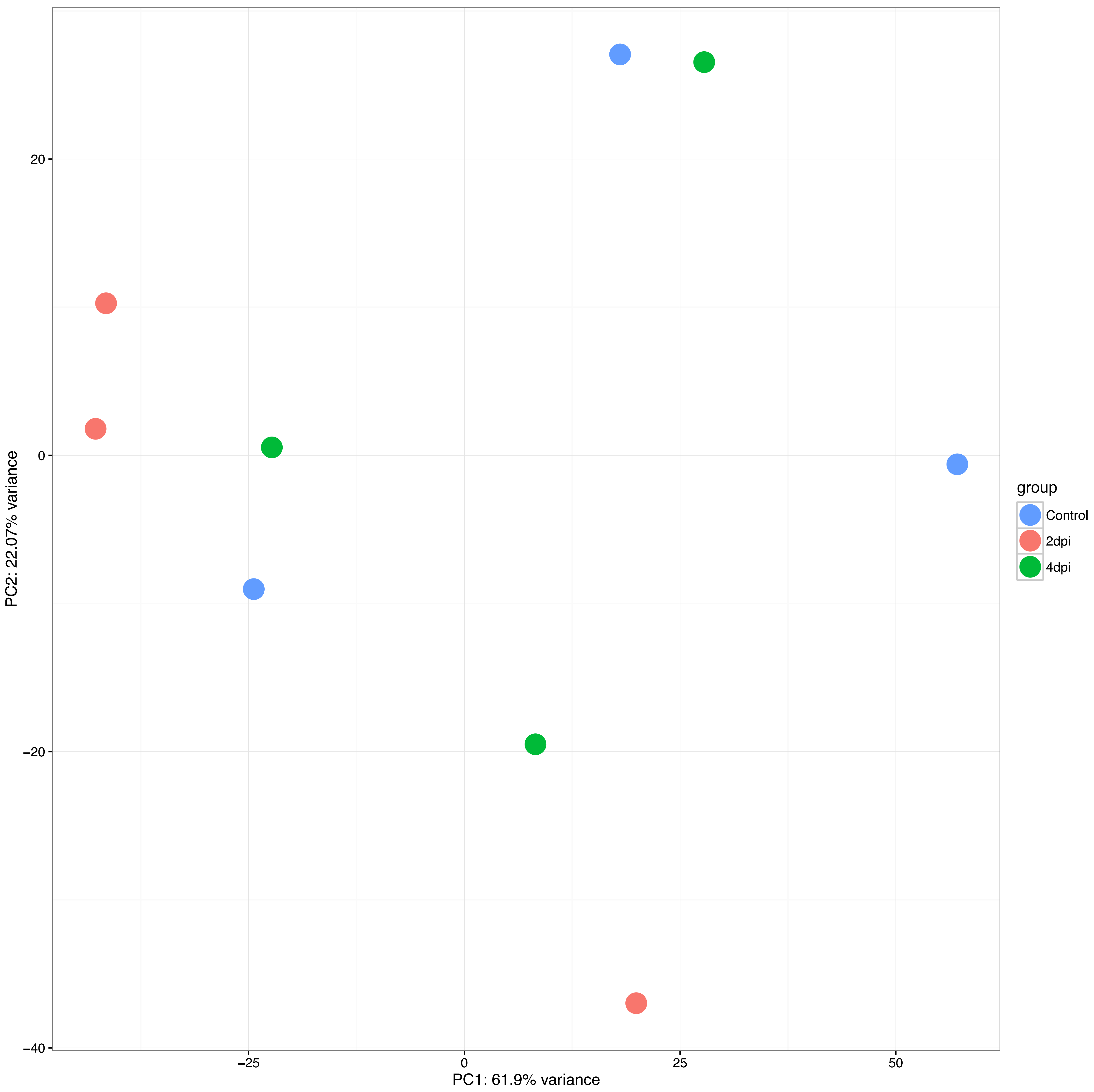

PC2: 22.07% variance
20
0
-20
-40
-25
0
25
50
PC1: 61.9% variance
group
Control
2dpi
4dpi

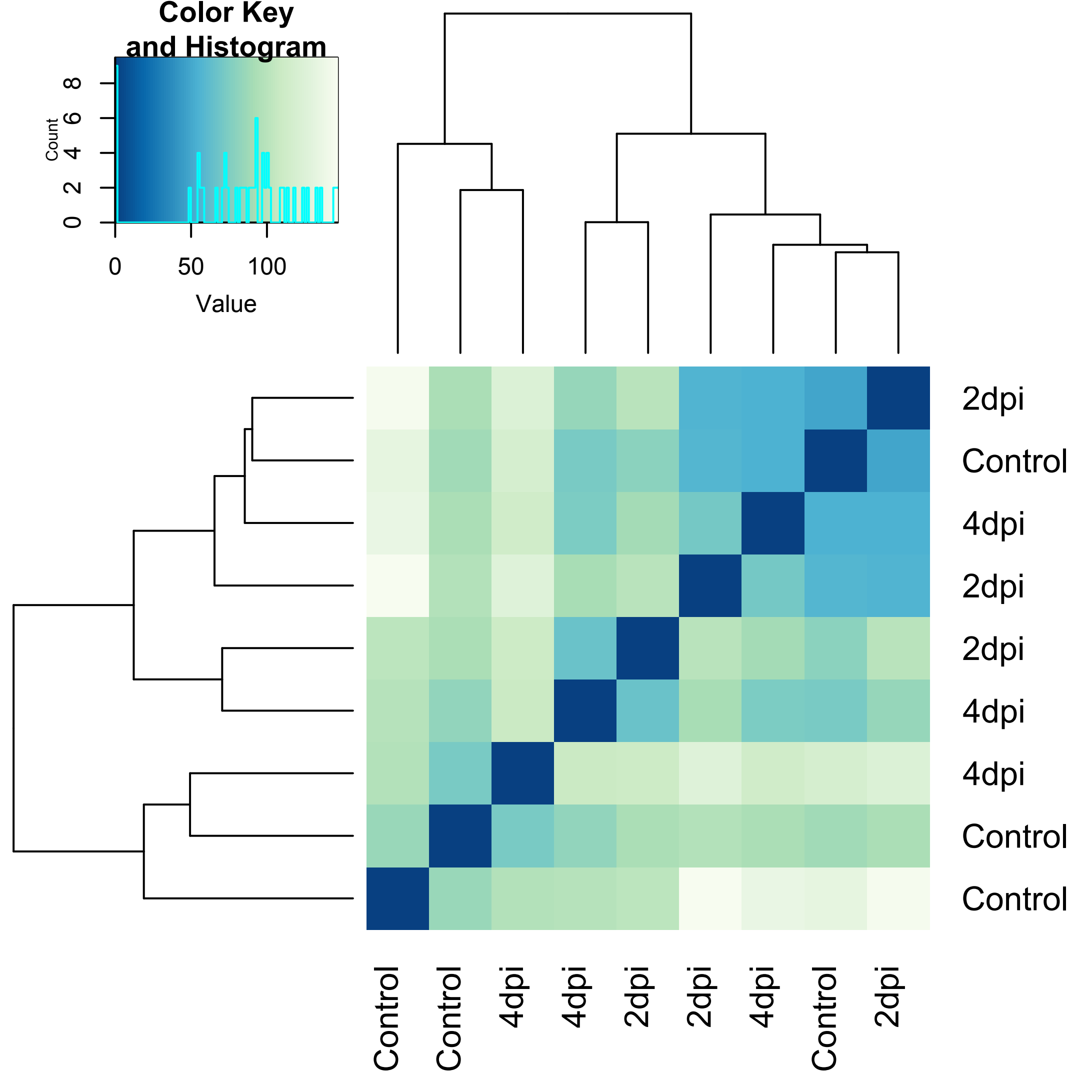
Color Key
and Histogram
Count
0 2 4 6 8
0 50 100
Value
2dpi
Control
4dpi
2dpi
2dpi
4dpi
4dpi
Control
Control
Control
Control
4dpi
4dpi
2dpi
2dpi
4dpi
Control
2dpi

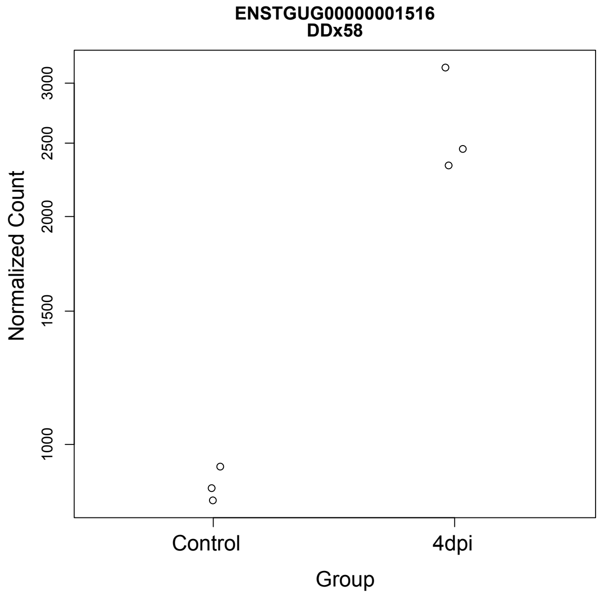
ENSTGUG00000001516
DDx58
Normalized Count
3000
2500
2000
1500
1000
Control
4dpi
Group

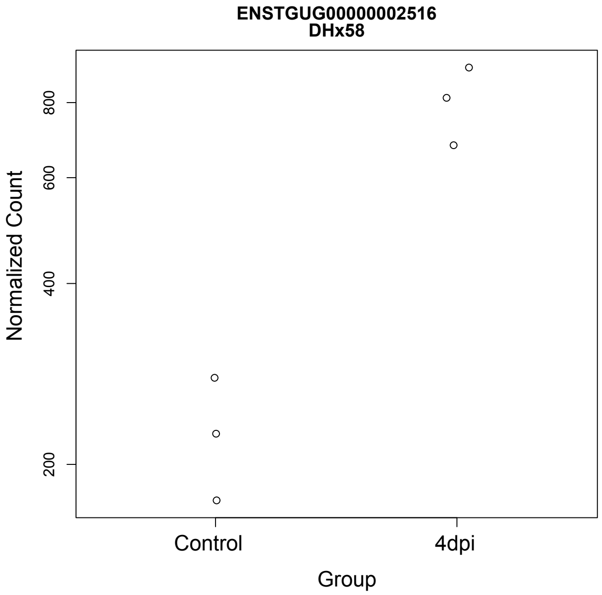
ENSTGUG00000002516
DHx58
Normalized Count
800
600
400
200
Control
4dpi
Group

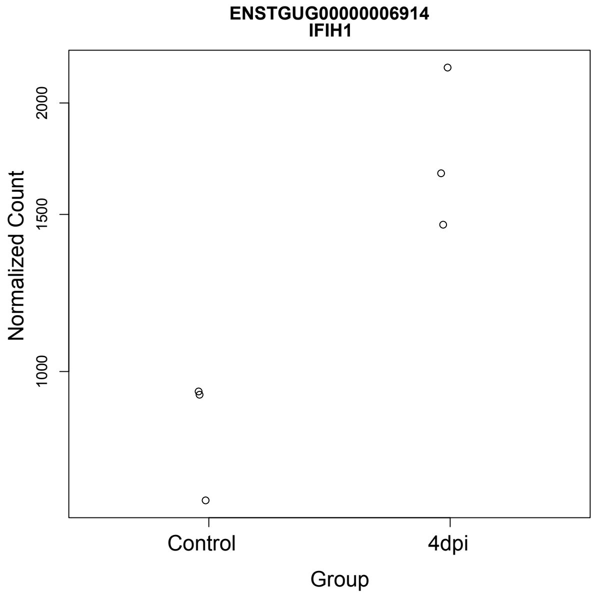
ENSTGUG00000006914
IFIH1
Normalized Count
2000
1500
1000
Control
4dpi
Group

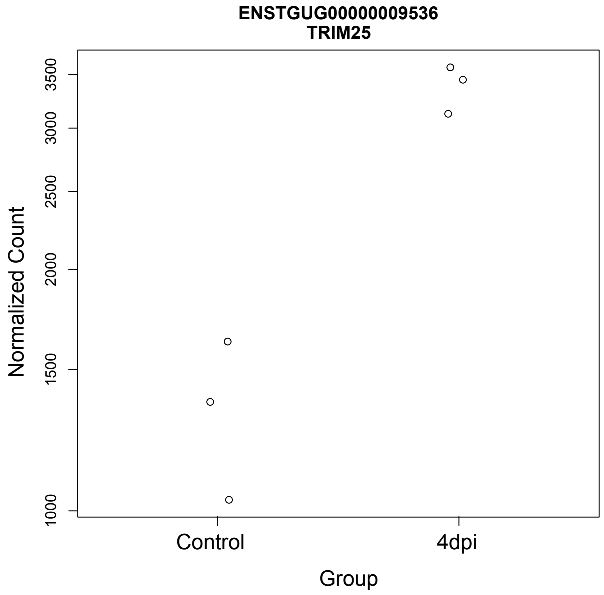
ENSTGUG00000009536
TRIM25
Normalized Count
3500
3000
2500
2000
1500
1000
Control
4dpi
Group

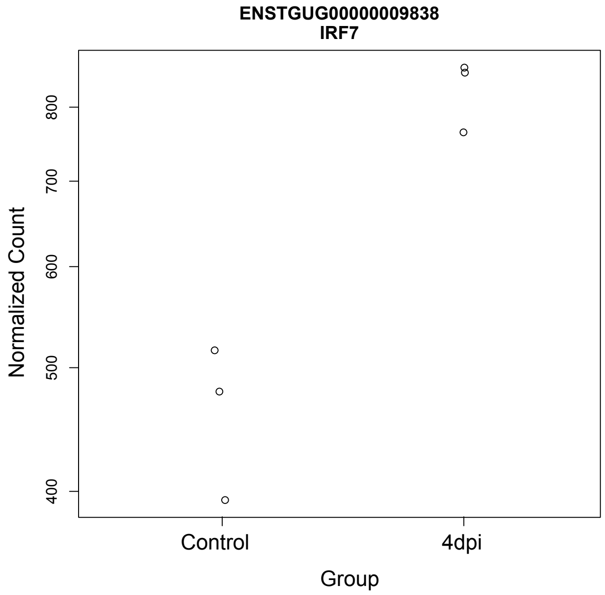
ENSTGUG00000009838
IRF7
Normalized Count
800
700
600
500
400
Control
4dpi
Group
